# Integrative Analysis Identifies Genetic Variants Associated With Autoimmune Diseases Affecting Putative MicroRNA Binding Sites

**DOI:** 10.3389/fgene.2018.00139

**Published:** 2018-04-26

**Authors:** Rodrigo C. de Almeida, Vinícius S. Chagas, Mauro A. A. Castro, Maria L. Petzl-Erler

**Affiliations:** ^1^Human Molecular Genetics Laboratory, Department of Genetics, Federal University of Paraná, Curitiba, Brazil; ^2^Molecular Epidemiology, Department of Biomedical Data Sciences, Leiden University Medical Center, Leiden, Netherlands; ^3^Bioinformatics and Systems Biology Laboratory, Federal University of Paraná, Curitiba, Brazil

**Keywords:** microRNA, SNP, autoimmunity, eQTL, GWAS, data integration

## Abstract

Genome-wide and fine mapping studies have shown that more than 90% of genetic variants associated with autoimmune diseases (AID) are located in non-coding regions of the human genome and especially in regulatory sequences, including microRNAs (miRNA) target sites. MiRNAs are small endogenous noncoding RNAs that modulate gene expression at the post-transcriptional level. Single nucleotide polymorphisms (SNPs) located within the 3′ untranslated region of their target mRNAs (miRSNP) can alter miRNA binding sites. Yet, little is known about their effect on regulation by miRNA and the consequences for AID. Conversely, it is well known that two or more AID may share part of their genetic background. Here, we hypothesized that miRSNPs could be associated with more than one AID. To identify miRSNPs associated with AID, we integrated results from three different prediction tools (Polymirts, miRSNP, and miRSNPscore) using a naïve Bayes classifier approach to identify miRSNPs predicted to affect binding sites of miRNAs. Further, to detect miRSNPs associated with two or more AID, we integrated predictions with summary statistics from 12 AID studies. In addition, to prioritize miRSNPs, miRNAs and AID-associated target genes, we used public expression quantitative trait locus (eQTL) data and mRNA-seq and small RNA-seq data. We identified 34 miRNSPs associated with at least two AID. Furthermore, we found 86 miRNAs predicted to target 18 of the associated gene's mRNAs. Our integrative approach revealed new insights into miRNAs and AID associated target genes. Thus, it helped to prioritize AID noncoding risk SNPs that might be involved in the causal mechanisms, providing valuable information for further functional studies.

## Introduction

Genome-wide association studies (GWAS) and fine mapping studies have identified approximately 250 loci associated with autoimmune disease (AID), and some of these loci are shared between two or more diseases (Ricaño-Ponce and Wijmenga, 2013). The recent results of fine mapping studies performed by the Immunochip platform, showed that the majority of the associated variants are located in non-coding regions, especially in regulatory sequences such as microRNAs (miRNAs) target sites (Ricaño-Ponce and Wijmenga, 2013).

MiRNAs are small molecules about 22 nt long that act by imperfect base-paring to 3′ untranslated regions (3′ UTR) of target messenger RNAs (mRNAs), leading to translation repression, degradation of the mRNA, or both (Bartel, 2009). MiRNAs negatively regulate their target mRNAs primarily through Watson and Crick base-pairing interactions, and the seed sequence located at positions 2–8 within the miRNA sequence is key for this action. Single nucleotide polymorphisms (SNPs) within the seed site, or in the target mRNA at sites complementary to miRNA seed sites, referred to as miRSNPs, may reduce effectiveness or abolish miRNA-mediated repression (Saunders et al., 2007), which could have functional consequences for complex diseases (Sethupathy and Collins, 2008), including AID (Hrdlickova et al., 2014).

In addition, it has been recently shown that numerous miRSNPs have expression quantitative trait loci (eQTL) effect on GWAS reported genes (Võsa et al., 2015). Moreover, the number of miRSNPs reported to be associated with human diseases increased in the past few years (Wei et al., 2012; Ghanbari et al., 2015; Stegeman et al., 2015; Cipolla et al., 2016), therefore, several *in silico* approaches has been developed to identify the potential impact of these polymorphisms in miRNA target genes (reviewed in Moszynska et al., 2017). Nevertheless, a systematically approach that integrates predictions from different miRSNPs algorithms has not yet been developed.

Here, we hypothesized that miRSNPs could be associated with more than one AID and that a comprehensive analysis of miRSNPs could identify additional still unknown risk variants. To investigate genetic associations of miRSNPs with AID, we applied an approach that integrates information provided by different algorithms, which uses several different data types. First, we used a naïve Bayes classifier to integrate results from three major prediction tools. Next, we intersected these results with summary statistics from GWAS of 12 common autoimmune diseases (AID), where 10 of these studies used the high-density platform Immunochip (Cortes and Brown, 2011). Moreover, by integrating small and mRNA-seq with eQTL public data, we were able to prioritize miRSNPs, miRNAs, and AID associated target genes. The results of our analyses provide valuable information for further functional studies.

## Materials and methods

### Data integration

In order to find SNPs predicted to have an effect on miRNA binding sites (miRSNPs) results from three different prediction tools were integrated:

(1) PolymiRts v.3.0 (http://compbio.uthsc.edu/miRSNP/; Bhattacharya et al., 2014); (2) miRNASNP2 (http://www.bioguo.org/miRNASNP2; Liu C. et al., 2012); and (3) miRSNPscore (http://www.bigr.medisin.ntnu.no/mirsnpscore; Thomas et al., 2011).

PolymiRTS v3.0 (accessed 15 Feb 2016) uses dbSNP v137, miRBase v20, and TargetScan (Friedman et al., 2009) algorithms to predict miRNA binding sites. To predict efficacy of targeting, this algorithm considers two major features, the probability of targeting conservation and the perfect match between the target and the seed sequence on the miRNA, that is, the at least 7 nt long canonical seed region (7mer-A1, 7mer-m8, or 8mer).

miRNASNP2 (accessed 15 Feb 2016) integrates data from miRBase v19, dbSNP v137 and uses two prediction tools: TargetScan and the miRanda (John et al., 2004) algorithm with stringent 2–8 nt pairing for miRNA binding site prediction. MiRanda assumes strict complementarity between nucleotides 2 and 8 and uses a score of alignment quality and free energy for miRNA binding site identification.

MirSNPscore uses miRBase v16, SNP data from HapMap 3 Project, and its own score algorithm to miRNA-biding site predictions. This tool uses haplotype information to calculate a score that corresponds to the probability of the SNP interfering with miRNA-binding.

To integrate results from these tools, a naïve Bayesian approach, adapted from previously successful data integration strategies (von Mering et al., 2005; Szklarczyk et al., 2017), was used. A naïve Bayes classifier was implemented in R (R Development Core Team, 2017) and a combined score was calculated for each miRSNP under the assumption of independence for the various data sources (data harmonization was obtained by z-score transformation of the results from each prediction algorithm):

(1) $$\begin{array}{l} \text{S}=1-\underset{\text{i}}{\prod }\left(1-{\text{S}}_{\text{i}}\right) \end{array}$$

MiRSNPs with naïve Bayes combined (NBC) score >0.7 were considered candidates to affect the miRNA-binding site.

### Summary statistics from autoimmune diseases (immunochip studies)

To investigate whether miRSNPs are associated with AID, we downloaded summary statistics for 12 diseases available at the Immunobase database (https://www.immunobase.org/, accessed 16 May 2016) and integrated them with the naïve Bayes classifier results. Cohort participants and studies are shown in Table 1. Of these studies, 10 used high-density genotype array Immunochip and two used the first generation of chip arrays (Illumina HumanHapMap 5, Illumina HumanHapMap3 and Affymetrix Gene Chip 500). Quality control procedures are described in detail in the original papers of each study (references are cited in Table 1).

**Table 1:** Cohorts description.

**Disease**	**Cases**	**Controls**	**References**	**Immunochip**
Crohn's disease	6,333	15,056	Franke et al., 2010	No (Meta-analysis)
Crohn's disease	5,937	8,043	Jostins et al., 2012	Yes
Ulcerative colitis	6,687	19,718	Anderson et al., 2011	No (Meta-analysis)
Ulcerative colitis	6,945	13,727	Jostins et al., 2012	Yes
Celiac disease	12,041	12,228	Trynka et al., 2011	Yes
Autoimmune thyroid disease	2,747	9,364	Cooper et al., 2008	Yes
Juvenile Idiopathic arthritis	2,816	13,056	Hinks et al., 2013	Yes
Multiple sclerosis	14,498	24,091	Beecham et al., 2013	Yes
Rheumatoid arthritis	11,475	15,870	Eyre et al., 2012	Yes
Systemic lupus erythematosus	5,201	9,066	Bentham et al., 2015	Yes
Type 1 diabetes	6,670	9,416	Onengut-Gumuscu et al., 2015	Yes
Primary biliary cirrhosis	2,861	8,514	Liu J. Z. et al., 2012	Yes

To capture also SNPs in linkage disequilibrium (LD) that were not reported as associated by the study, we used LDlink tool (Machiela and Chanock, 2014) using data from the 1000 Genomes Project phase 3 (Abecasis et al., 2012) with standard parameters (*r*^2^ ≥ 0.8 and D' = 1) and the CEU population as reference panel.

For our purpose, only miRSNPs that either had genome-wide threshold *P*-value (*P* < 5 × 10^−8^) or were in strong LD (*r*^2^ > 0.8) with a significantly associated SNP but not evaluated in the study, were considered as candidates. In addition, only miRSNPs associated with at least two AID were considered for further analysis.

### eQTL data integration

eQTL data was acquired from three different databases, BloodeQTLBrowser (http://genenetwork.nl/bloodeqtlbrowser/; Westra et al., 2013), the Geuvadis project (Lappalainen et al., 2013), and the GTEx v6 portal (http://www.gtexportal.org/home; Lonsdale et al., 2013).

BloodeQTLBrowser contains data from peripheral blood of 5,311 individuals obtained with microarray chips. The Geuvadis project (Lappalainen et al., 2013) used RNA-seq data from 465 lymphoblastoid cell lines (LCL) from 5 populations of the 1000 Genomes Project: the CEPH (CEU), Finns (FIN), British (GBR), Toscani (TSI), and Yoruba (YRI) to perform its eQTL analysis. Data from GTEx v6 portal (Lonsdale et al., 2013) derived from 44 different tissues in 7,051 RNA-seq samples.

BloodeQTLBrowser and Geuvadis data were filtered using false discovery rate (FDR) correction at the 0.05 level, which resulted in 514,000 cis-eQTLs from BloodeQTL and 7,714 cis-eQTLs from the Geuvadis project. The GTEx v6 data used a FDR ≤ 0.05, containing 27,159 unique cis-eQTL genes.

### Small RNA-seq, mRNA-seq and genotype data from the GEUVADIS and the 1000 genomes projects

mRNA-seq and small RNA-seq data from 465 LCL were downloaded from the Geuvadis Project (Lappalainen et al., 2013). After matching samples that have both the miRNA and the mRNA profiles, 321 RNA-seq samples remained, which were further log_2_ normalized. Further, genotype data were downloaded from the 1000 Genomes Project (Abecasis et al., 2012). After matching with individuals from the Geuvadis project, 309 samples with genotype and RNA-seq (small RNA and mRNA) data were used for Pearson correlation analysis between the expression levels of the miRNAs and their mRNAs targets, where *P* < 0.05 was considered the limit for statistical significance.

### Experimentally validated miRNA-target interactions databases

Experimentally validated miRNA target sites were downloaded from miRTarBase v7.0 (Chou et al., 2016; http://mirtarbase.mbc.nctu.edu.tw/php/index.php) and Tarbase v7 (Vlachos et al., 2015; under authorization on http://diana.imis.athena-innovation.gr/DianaTools/index.php?r=tarbase/index). These databases contain hundreds of thousands of published experimentally manually curated validated miRNA-target interactions. These databases include data generated by the main types of functional experiments currently used to validate miRNA-mRNA interaction, such as, western blot, microarray, luciferase report assays and high-throughput sequencing of immunoprecipitated RNAs after cross-linking (CLIP-seq), submitted by many other researchers.

## Results

### Data integration showed that autoimmune diseases share associations with miRSNPs

To identify SNPs that affect the binding site of miRNAs (miRSNPs) in genes associated with AID, we implemented a naïve Bayes classifier approach to integrate different miRSNPs prediction tools and further intersection with summary statistics from GWAS high density genotyped data (Immunochip).

We integrated three miRSNPs prediction tools with summary statistics from 12 GWAS of AID available in the Immunobase database (Table 1).

However, even studies using a dense genotyping custom-made array designed to fine-map immune-related diseases, such as the Immunochip, may still miss associations due to differences in linkage disequilibrium (LD) pattern between populations, or because of differences of quality control steps. To minimize this bias, we also included miRSNPs that were in high LD (*r*^2^ ≥ 0.8 and D' = 1 in the CEU population Abecasis et al., 2012) with the most associated SNP of the corresponding target gene reported by each study (Supplementary Table 1).

Additionally, to identify miRSNPs that might affect expression levels of the reported disease-associated genes, we intersected our results with publicly available eQTL data from the GTEx project (Lonsdale et al., 2013), Blood eQTL Browser (Westra et al., 2013), and the Geuvadis project (Lappalainen et al., 2013) (Supplementary Table 2).

We found 34 miRSNPs that may alter the binding sites of 86 miRNAs predicted to target mRNAs of 18 genes (Table 2). Of these, 28 miRSNPs displayed an eQTL effect on 13 target genes in several tissues (Table 2). Moreover, from the 34 miRNSPs, four (rs1054037, rs2070197, rs727088, rs7444) had been reported as the most associated SNPs by the original studies (*P* < 5 × 10^−8^) (Table 2). The remaining 30 miRSNPs were in strong LD with the most associated SNP reported, but were not evaluated by the study. For instance, two miRSNPs (rs60474474 and rs45450798), both located in the 3′ UTR of *PTPN2*, were not reported in any of the GWAS. However, these two miRSNPs display maximum possible LD (*r*^2^ = 1 and D' = 1) (Supplementary Table 1) with the most associated SNP reported for five AIDs inflammatory bowel disease (IBD), Crohn's disease (CD), type 1 diabetes (T1D), celiac disease (CeD), juvenile idiopathic arthritis (JIA) (Table 2). Both miRSNPs were predicted to alter miRNA binding sites in the 3′ UTR of the *PTPN2* mRNA: miRSNP rs60474474 disrupts the binding site of miR-4290 (NBC Score = 0.99) and miRSNP rs45450798 creates a new binding site for miR-4531 (NBC Score = 0.73) (Table 2).

**Table 2:** MiRSNPs associated with autoimmune disease.

**miRSNP**	**chr**	**pos_bp(hg38)**	**Ref. allele**	**Minor allele**	**MAF**	**Disease**	***P*-value**	**OR**	**Target gene**	**miRNA**	**Effect on miRNA**	**NBS**	**eQTL (effect direction)**
rs7559479	2	102452327	A	G	0.22	Crohn's disease	2.2e-10	1.15	*IL18RAP*	hsa-miR-3156-3p	C	0.79	Yes (up)
						Celiac disease	1.4e-16	1.19		hsa-miR-4301	C	0.86	
						Inflammatory bowel disease	NA	NA		hsa-miR-136	C	0.92	
rs7603250	2	102452374	A	T	0.22	Crohn's disease	1.6e-10	0.8	*IL18RAP*	hsa-miR-455-3p	C	0.94	Yes (up)
						Inflammatory bowel disease	1.8e-09	0.8					
						Celiac disease	NA	NA					
rs3732421	3	119431242	A	G	0.16	Multiple sclerosis	3.5e-12	0.85	*TMEM39A*	hsa-miR-449b-3p	D	0.72	No
						Primary biliary cirrhosis	1.7e-11	0.72		hsa-miR-4691-3p	D	0.82	
										hsa-miR-449b	D	0.92	
rs57271503	3	119525746	G	A	0.16	Multiple sclerosis	4.1e-15	0.84	*CD80*	hsa-miR-769-5p	C	0.71	No
						Primary biliary cirrhosis	9.4e-13	0.71		hsa-miR-4802-5p	C	0.80	
										hsa-miR-769-5p	C	0.71	
**rs1054037**	4	102631552	T	C	0.49	**Primary biliary cirrhosis**	8.3e-10	0.82	*MANBA*	hsa-miR-660	D	0.90	Yes (down/up)
						Multiple sclerosis	NA	NA		hsa-miR-5591-3p	D	0.95	
										hsa-miR-660-5p	D	0.82	
										hsa-miR-7151-5p	C	0.71	
										hsa-miR-3686	C	0.94	
rs4013	4	102631656	T	C	0.51	Primary biliary cirrhosis	NA	NA	*MANBA*	hsa-miR-4742-3p	C	0.76	Yes (down/up)
						Multiple sclerosis	NA	NA		hsa-miR-4778-5p	C	0.91	
										hsa-miR-630	C	0.82	
rs1054029	4	102631896	A	G	0.51	Primary biliary cirrhosis	NA	NA	*MANBA*	hsa-miR-124-5p	D	0.80	Yes (down/up)
						Multiple sclerosis	NA	NA		hsa-miR-4255	D	0.99	
										hsa-miR-4766-5p	D	0.74	
										hsa-miR-124	D	0.90	
										hsa-miR-33a	D	0.77	
rs39602	5	97028750	G	C	0.41	Ankylosing spondylitis	NA	NA	*LNPEP*	hsa-miR-6800-5p	D	0.99	Yes (down)
						Crohn's disease	6.9e-11	1.1					
						Juvenile idiopathic arthritis	NA	NA					
**rs2070197**	7	128948946	T	C	0.1	**Systemic lupus erythematosus**	3.0e-40	1.7	*IRF5*	hsa-miR-3136-3p	D	0.91	Yes (up)
						Primary biliary cirrhosis	1.8e-18	1.5		hsa-miR-7155-3p	D	0.89	
rs10114470	9	114785492	C	T	0.33	Crohn's disease	1.5e-15	1.1	*TNFSF15*	hsa-miR-376a-3p	D	0.84	Yes (up)
						Inflammatory bowel disease	1.5e-16	0.8		hsa-miR-4753-5p	D	0.74	
rs3088081	9	136375697	A	G	0.42	Inflammatory bowel disease	1.9e-22	0.8	*SNAPC4*	hsa-miR-3661	C	0.80	yes (down)
						Crohn's disease	2.1e-17	0.8					
						Ulcerative colitis	1.0e-11	0.8					
rs9943	13	39752145	A	G	0.34	Juvenile idiopathic arthritis	NA	NA	*COG6*	hsa-miR-628-5p	C	0.98	Yes (down)
						Rheumatoid arthritis	NA	NA					
rs3839999	13	99385548	AT	A	0.22	Crohn's disease	NA	NA	*UBAC2*	hsa-miR-365a-3p	C	0.93	No
						Inflammatory bowel disease	NA	NA					
rs907091	17	39765489	C	T	0.51	Primary biliary cirrhosis	1.0e-10	0.79	*IKZF3*	hsa-miR-4497	D	0.97	Yes (down)
						Type 1 diabetes mellitus	NA	NA		hsa-miR-3649	C	0.85	
						Ulcerative colitis	8.1e-09	0.8		hsa-miR-4518	C	0.92	
						Inflammatory bowel disease	3.3e-13	0.8		hsa-miR-330-5p	C	0.99	
										hsa-miR-518c	C	0.86	
										hsa-miR-4314	C	0.84	
										hsa-miR-326	C	0.99	
										hsa-miR-3192	C	0.73	
rs16940681	17	45834793	G	C	0.24	Primary biliary cirrhosis	NA	NA	*CRHR1*	hsa-miR-6740-5p	D	0.84	Yes (up)
						Type 1 diabetes mellitus	NA	NA					
rs2316765	17	45835088	T	C	0.24	Primary biliary cirrhosis	NA	NA	*CRHR1*	hsa-miR-3192	D	0.73	Yes (up)
						Type 1 diabetes mellitus	NA	NA		hsa-miR-30c-2-3p	D	0.97	
										hsa-miR-30c-1-3p	D	0.90	
rs878886	17	45835124	C	G	0.24	Primary biliary cirrhosis	NA	NA	*CRHR1*	hsa-miR-4685-5p	D	0.96	Yes (up)
						Type 1 diabetes mellitus	NA	NA		hsa-miR-1915-3p	D	0.71	
										hsa-miR-3918	D	0.86	
										hsa-miR-7160-3p	D	0.76	
rs878887	17	45835216	C	T	0.24	Primary biliary cirrhosis	NA	NA	*CRHR1*	hsa-miR-198	C	0.85	Yes (up)
						Type 1 diabetes mellitus	NA	NA		hsa-miR-3186-5p	D	0.88	
										hsa-miR-136	C	0.92	
rs878888	17	45835269	A	G	0.24	Primary biliary cirrhosis	NA	NA	*CRHR1*	hsa-miR-5708	C	0.70	Yes (up)
						Type 1 diabetes mellitus	NA	NA		hsa-miR-1226-5p	D	0.72	
rs4640231	17	45835420	G	C	0.24	Primary biliary cirrhosis	NA	NA	*CRHR1*	hsa-miR-6841-5p	D	0.81	Yes (up)
						Type 1 diabetes mellitus	NA	NA		hsa-miR-6755-5p	D	0.77	
rs4482334	17	45835464	T	C	0.24	Primary biliary cirrhosis	NA	NA	*CRHR1*	hsa-miR-6890-5p	D	0.93	Yes (up)
						Type 1 diabetes mellitus	NA	NA		hsa-miR-6742-5p	D	0.94	
										hsa-miR-4722-5p	D	0.77	
										hsa-miR-6796-5p	D	0.75	
										hsa-miR-4459	D	0.80	
rs12373168	17	45846971	A	C	0.24	Primary biliary cirrhosis	NA	NA	*SPPL2C*	hsa-miR-33b-3p	D	0.87	No
						Type 1 diabetes mellitus	NA	NA		hsa-miR-519e-3p	D	0.87	
rs60474474	18	12792737	C	T	0.14	Inflammatory bowel disease	1.4e-10	1.1	*PTPN2*	hsa-miR-4290	D	0.99	No
						Crohn's disease	2.2e-12	1.2					
						Type 1 diabetes mellitus	NA	NA					
						Celiac disease	NA	NA					
						Juvenile idiopathic arthritis	NA	NA					
rs45450798	18	12792941	C	G	0.14	Inflammatory bowel disease	2.0e-10	0.8	*PTPN2*	hsa-miR-4531	C	0.73	No
						Crohn's disease	2.9e-12	0.8					
						Type 1 diabetes mellitus	NA	NA					
						Celiac disease	NA	NA					
						Juvenile idiopathic arthritis	NA	NA					
rs9950174	18	69846569	T	C	0.53	Psoriasis	NA	NA	*CD226*	hsa-miR-5189-3p	D	0.85	Yes (up)
**rs727088**	18	69863203	G	A	0.53	**Inflammatory bowel disease**	4.6e-9	1.08	*CD226*	hsa-miR-513a-3p	D	0.75	Yes (up)
						**Ulcerative colitis**	1.9e-8	1.1		hsa-miR-181c	D	0.75	
						Psoriasis	NA	NA					
rs571689	19	48704297	C	T	0.47	Crohn's disease	7.3e-09	1.1	*FUT2*	hsa-miR-648	D	0.89	Yes (down)
						Type 1 diabetes mellitus	NA	NA		hsa-miR-552-3p	C	0.92	
rs570794	19	48704394	T	C	0.47	Crohn's disease	1.0e-08	0.8	*FUT2*	hsa-miR-4430	D	0.71	Yes (down)
						Type 1 diabetes mellitus	NA	NA		hsa-miR-1295b-5p	C	0.79	
										hsa-miR-4463	C	0.95	
										hsa-miR-1912	C	0.94	
rs507766	19	48705286	T	C	0.47	Crohn's disease	1.4e-08	0.8	*FUT2*	hsa-miR-136-5p	C	0.83	Yes (down)
						Type 1 diabetes mellitus	NA	NA		hsa-miR-675	C	0.80	
										hsa-miR-887	C	0.92	
										hsa-miR-410	C	0.73	
										hsa-miR-191	C	0.75	
rs506897	19	48705372	G	C	0.47	Crohn's disease	7.3e-09	1.1	*FUT2*	hsa-miR-4530	C	0.81	Yes (down)
						Type 1 diabetes mellitus	NA	NA					
rs503279	19	48705753	T	C	0.47	Crohn's disease	6.7e-09	0.8	*FUT2*	hsa-miR-675-3p	C	0.86	Yes (down)
						Type 1 diabetes mellitus	NA	NA					
rs1056441	20	63738996	C	T	0.28	Crohn's disease	5.4e-11	0.8	*LIME1*	hsa-miR-4745-3p	C	0.97	Yes (down)
						Inflammatory bowel disease	1.5e-15	0.8		hsa-miR-1538	C	0.97	
										hsa-miR-4467	C	0.87	
										hsa-miR-6770-3p	C	0.78	
										hsa-miR-3940-3p	D	0.82	
										hsa-miR-762	D	0.94	
**rs7444**	22	21622645	T	C	0.2	**Systemic lupus erythematosus**	1.8e-22	1.27	*UBE2L3*	hsa-miR-4741	D	0.90	Yes (up)
						Crohn's disease	1.3e-12	1.1		hsa-miR-4763-3p	D	0.89	
						Inflammatory bowel disease	6.9e-10	0.8		hsa-miR-3918	D	0.86	
										hsa-miR-1207-5p	D	0.92	
rs7445	22	21622758	C	T	0.19	Crohn's disease	6.3e-09	1.1	UBE2L3	hsa-miR-3064-5p	C	0.93	Yes (up)
						Inflammatory bowel disease	3.5e-10	1.1					
						Systemic lupus erythematosus	1.0e-12	1.27					

*NA, P-value was not available in the source study but this miRSNP is in linkage disequilibrium (r^2^ ≥ 0.8) with the most associated SNP reported; P-value obtained from each study. Bold: reported as associated in the original study. C, creates a binding site; D, disrupts a binding site; chr, chromosome; MAF, global minor allele frequency from the 1000 Genomes project; up, upregulated effect; down, downregulated effect; pos_bp, position in base pair; OR, odds ratio. OR was calculated based on the minor allele of the respective variant; eQTL, expression quantitative trait locus; NBC, naïve Bayes combined score*.

Among the investigated 12 diseases, Crohn's disease was the one that displayed more associated miRSNPs. In total, we found 16 miRSNPs associated with CD, affecting the binding sites of 31 miRNAs in the mRNAs of the CD-associated genes (Table 2).

The most significantly associated miRSNP (rs2070197) was associated with two diseases, systemic lupus erythematosus (SLE) (*P* = 3.04 × 10^−40^) and primary biliary cirrhosis (PBC) (*P* = 1.8 × 10^−18^) (Table 2). This miRSNP is located in the 3′ UTR of the *IRF5* gene and may disrupt the binding sites of two miRNAs (miR-3136-3p NBC Score = 0.91 and miR-7155-3p NBC Score = 0.89). Thus, it is an eQTL predicted to downregulate the expression of *IRF5* at least in LCL.

**Figure 1:**
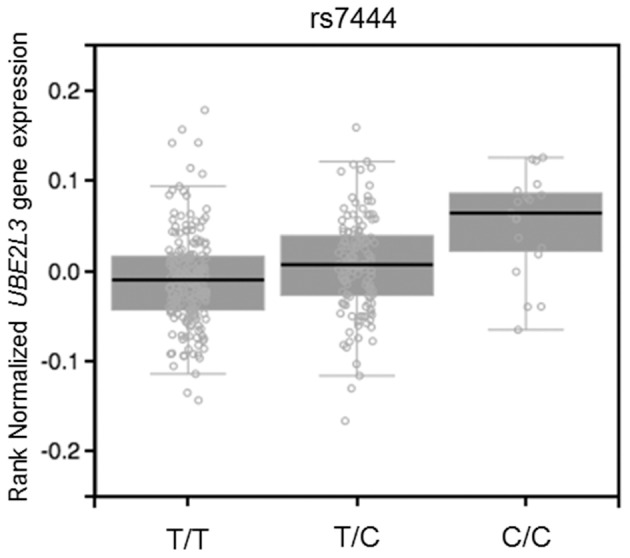
eQTL effect on whole blood of SNP rs7444 genotypes on expression of *UBE2L3* mRNA (*P* = 1.7 × 10^−7^, effect size = 0.16). Figure adapted from the Gtex database (https://www.gtexportal.org).

The miRSNP rs7444, which is in the 3′ UTR of the *UBE2L3* gene, was associated with three AIDs (SLE, CD, and IBD). This miRSNP was predicted to affect binding of four miRNAs (miR-4741 NBC Score = 0.9, miR-4763-3p NBC Score = 0.89, miR-3918 NBC Score = 0.86, miR-1207-5p NBC Score = 0.92) and is an eQTL that upregulates the target gene in whole blood (Figure 1).

Moreover, the gene *FUT2*, which was associated with three AID (CD, IBD, and T1D), had five miRSNPs affecting 13 miRNAs. These miRSNPs are eQTL with a downregulation effect on the target mRNAs in several tissues, including small intestine and whole blood (Table 2).

**Figure 2:**
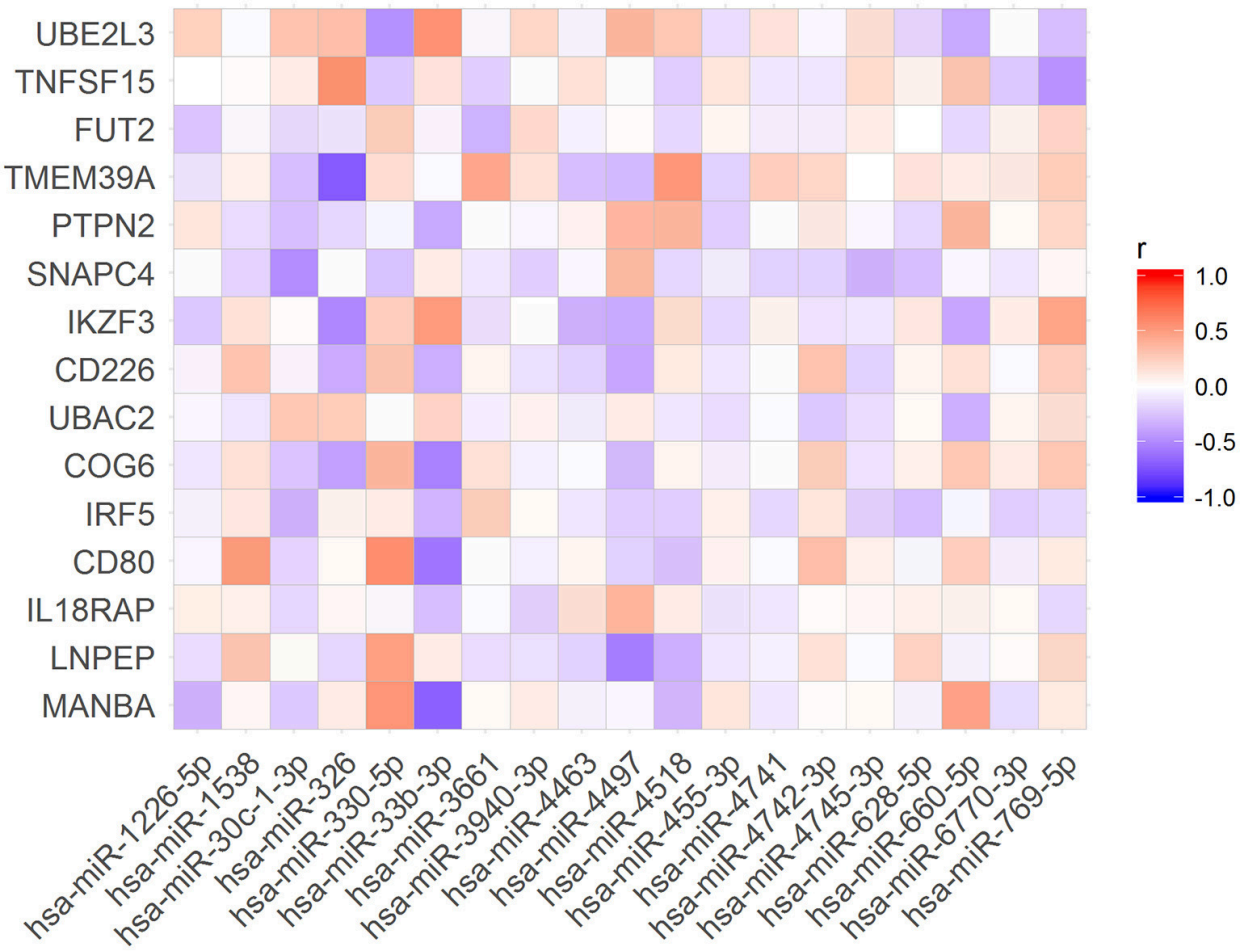
Pearson correlations between the expression levels of the miRNAs and their target mRNAs.

**Figure 3:**
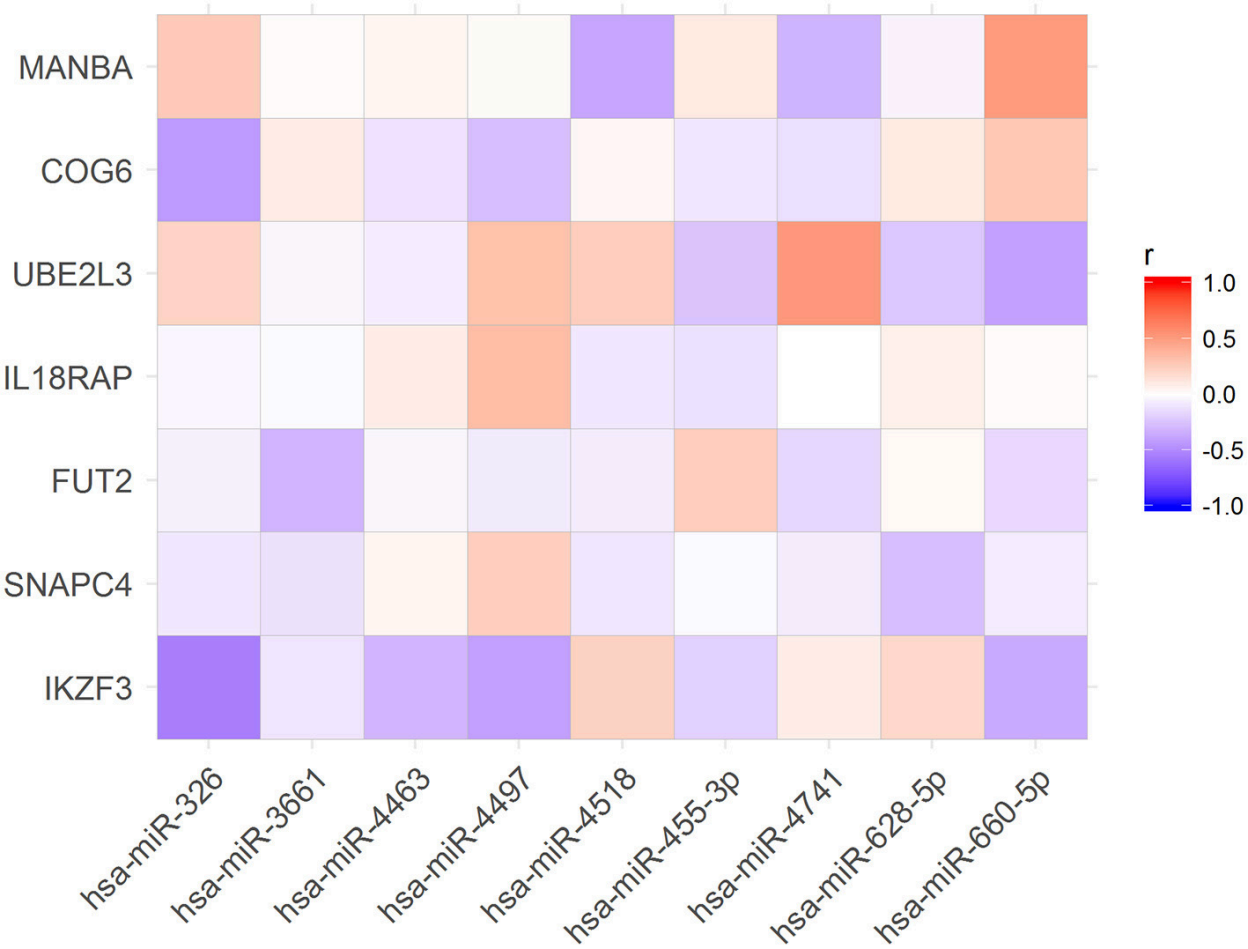
Expression levels of nine microRNA were correlated with expression levels of their target mRNAs as expected according with TargetScan and miRanda predictions.

To verify if the expression levels of miRNAs could be correlated with the mRNA levels of the associated target genes, we used public available RNA-seq data from the Geuvadis Project (Lappalainen et al., 2013). First, we matched all samples that had both, small RNA-seq and mRNA-seq data (*N* = 321 samples). Further, we performed Pearson correlation analysis of the expression of all miRNAs and the mRNA of their targets. Of the 86 miRNAs in our dataset (Table 2) we found 19 whose expression levels were correlated with those of 15 predicted targets (Figure 2). Nevertheless, only 9 of these 19 miRNAs were correlated with expression levels of their target genes in the same direction (up- or downregulated) corresponding to that predicted by the algorithms (Figure 3).

**Figure 4:**
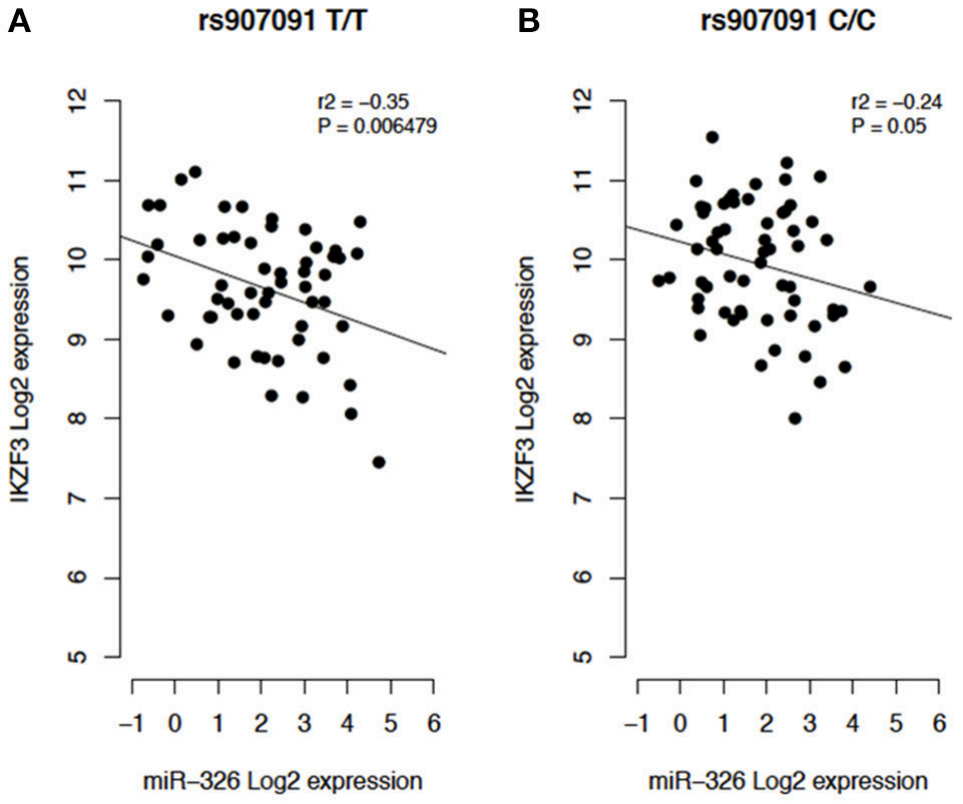
**(A)** Individuals homozygous for the protective allele T (T/T). **(B)** Individuals homozygous for the reference allele C (C/C).

**Figure 5:**
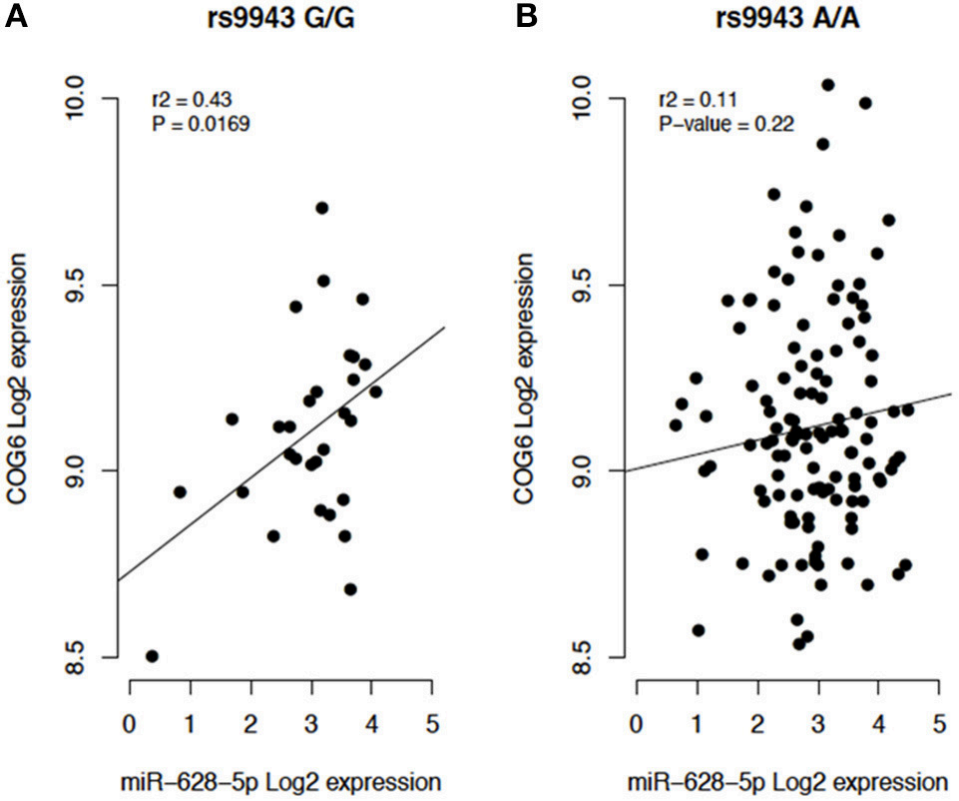
**(A)** Individuals homozygous for the risk allele rs9943 G (G/G). **(B)** Individuals homozygous for the reference allele A (A/A).

**Figure 6:**
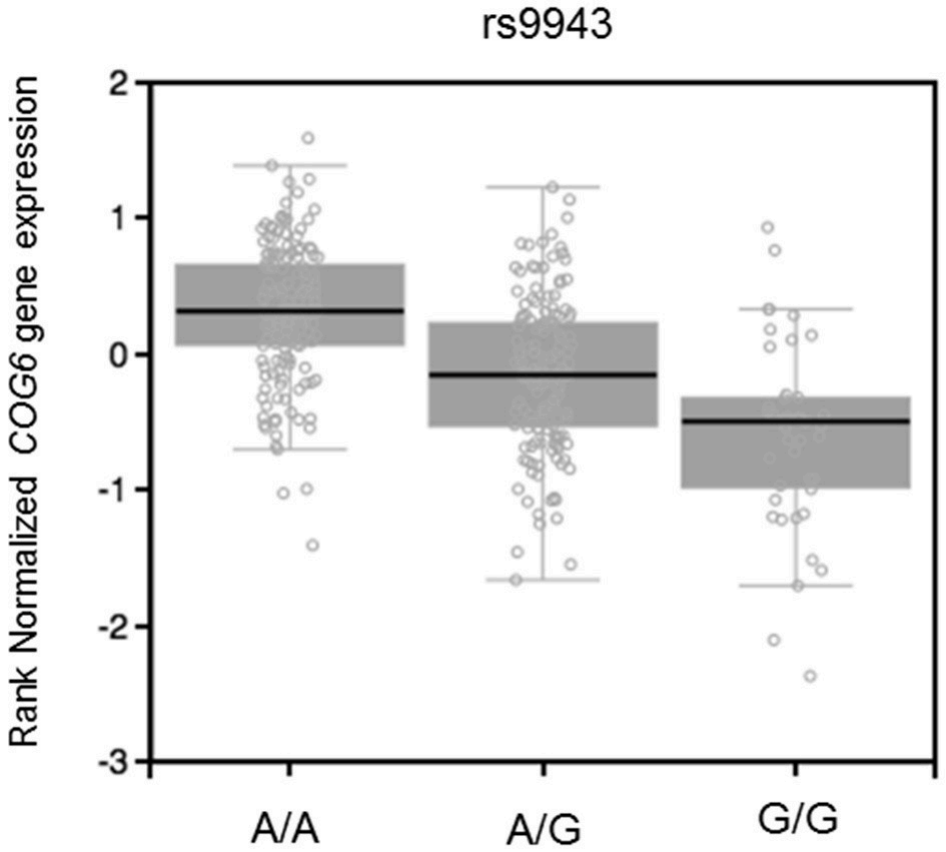
eQTL effect on muscle skeletal of rs9943 genotypes on expression of *COG6* mRNA (*P* = 2.0e-25, effect size = −0.53). Figure adapted from the Gtex database (https://www.gtexportal.org).

To investigate whether correlation of expression levels of these 9 miRNAs and their targets were dependent on the genotype, we extracted genotype data from the 1000 Genomes Project, and RNA-seq data (small RNA and mRNA) from the Geuvadis project. We then performed Pearson correlation analysis with only homozygous individuals for the minor and the reference alleles of each miRSNP. We found that expression levels of three miRNAs correlated with those of two target genes (*P* < 0.05) depending on the genotype (Figures 4–**6**). Nevertheless, when comparing the correlation between the mRNA and microRNA expression data between genotypes, the predictions of the miRSNP's effect on the expression of the target gene could not be clearly demonstrated. In the sample of individuals homozygous for the minor allele (T) of miRSNP rs907091 located in the 3′ UTR of *IKZF3*, expression of miR-326, and *IKZF3* were negatively correlated (*P* = 0.006479, *r*^2^ = −0.35) (Figure 4). This miRSNP is predicted to create a binding site for miR-326 with a high NBC Score (NBC Score = 0.99), which fits the negative correlation observed between expression of miR-326 and the *IKZF3* mRNA. Yet, although not significant, the correlation follows the same trend in the sample of individuals homozygous for allele C (Figure 4). Furthermore, the expression levels of miR-4518 and the *IKZF3* mRNA were positively correlated (*P* = 0.02, *r*^2^ = 0.2) in the presence of homozygosity for allele rs907091 T, which is predicted to create a binding site for miR-4518 in the 3′ UTR of that mRNA.

In addition, miRSNP rs9943 allele G, which is an eQTL associated with downregulation of the target gene *COG6* (Figure 6), was predicted to create a binding site for miR-628-5p. Notwithstanding, expression of this miRNA and of the mRNA of its target *COG6* are positively correlated (*P* = 0.01, *r*^2^ = 0.43) for genotype G/G but are not significantly correlated for genotype A/A (Figure 5). It should be noticed that data from the Geuvadis project derive from LCL only and may not reflect the expression pattern in cells relevant for pathogenesis of the AID. Altogether, these results reveal the complexity of inferences based on predictions and the importance of functional validation of the hypotheses.

Furthermore, in order to verify if the predictions of miRNA-target interactions had been experimentally validated, we integrated our results with data available in the DIANA-Tarbase v7 (Vlachos et al., 2015) and the MiRTarbase (Chou et al., 2016). We found two miRNA—target interactions (*UBAC2*—miR-365a-3p and *SNAPC4*—miR-3661) which were experimentally validated by the CLIP-seq method (Supplementary Table 3). Variants of both validated target genes are associated with IBD and CD (Table 2), and *SNAPC4* is also associated with UC. In addition, the rs3088081 miRSNP is an eQTL with a downregulation effect of gene *SNAPC4* in thyroid and skeletal muscle tissues (Supplementary Table 2).

## Discussion

We used an approach to integrate miRNAs targets predictions, GWAS summary statistics and RNA-seq publicly available data, to investigate whether SNPs associated with two or more AID may affect miRNAs binding sites. Furthermore, we integrated eQTL results with RNA sequencing data (miRNA-seq and mRNA-seq) to verify if the genotypes of the selected miRSNPs influence the expression levels of the miRNAs and the mRNAs of their predicted target genes.

We identified 34 miRNSPs that may affect the binding sites of 86 miRNAs in 18 target genes, of which 30 were not previously reported by any of the original GWAS.

The gene *PTPN2* displayed highest number of associated diseases, five in total. This gene had two miRSNPs in its 3′ UTR. The risk allele (T) of miRSNP rs60474474 is predicted to disrupt the binding of miR-4290. On the other hand, the miRSNP rs45450798 also located in the 3′ UTR of *PTPN2*, has its protective allele (G) predicted to create a binding site for miR-4531. Tyrosine-protein phosphatase non-receptor type 2 (PTPN2) attenuates JAK/STAT signaling, among other effects. In mice, PTPN2 deficiency results in perturbations of T cell tolerance and enhanced T cell and B cell responses, resulting in severe inflammatory disease and autoimmunity (Wiede et al., 2017). Although we could not find evidence of an eQTL effect for these miRSNPs in any of the public databases, it has been shown that non-coding SNPs repressing *PTPN2* are associate with several immune related diseases, including T1D (Bottini et al., 2004), RA (Begovich et al., 2004), and CD (Festen et al., 2011). This agrees with our findings, once deregulation of PTPN2 expression by the creation of a miRNA binding site by the risk allele could eventually reduce expression PTPN2, favoring autoimmune disease. Additionally, miR-4290 is present in exosomes (Leidinger et al., 2014) but not in any blood cell type or whole blood, suggesting that ectopic or increased expression of this miRNA could be a candidate biomarker for immune-related diseases.

eQTL mapping studies have shown that SNPs associated with complex diseases, detected in GWAS, are more likely to be an eQTL compared to non-associated SNPs (Nicolae et al., 2010). In autoimmune disease it has been shown that approximately 12% of causal non-coding SNPs are eQTL (Farh et al., 2015). In addition, eQTL data integration with prediction of miRSNPs can help to link causal non-coding disease variants to specific genes (Võsa et al., 2015). We found that 28 miRSNPs associated with AID were eQTLs for their target genes. Interestingly, these results agree with results obtained with our naïve Bayes classifier predictions. One interesting example of this scenario is the miRSNP rs7444. This miRSNP is an eQTL that may upregulate *UBE2L3* (the gene that encodes the ubiquitin-conjugating enzyme E2 L3) and is predicted to disrupt the binding of four miRNAs to the mRNA of this target gene. The risk allele (C) disrupts binding of miRNAs with the *UBE2L3* mRNA, resulting in overexpression of *UBE2L3*, which is in perfect agreement with the eQTL results. Although *UBE2L3* polymorphisms have been associated with seven AID (Ricaño-Ponce and Wijmenga, 2013), after applying our approach and considering the stringent genome-wide *P*-value threshold (*P* < 5 × 10^−8^), we found three AID (SLE, CD, and IBD) sharing association with *UBE2L3*, indicating deregulation of miRNAs pathways at least in these three diseases. *UBE2L3* participates of ubiquitination of the NF-κB precursor (Whiteside, 1995), a major transcription factor for genes involved in inflammation and immune responses. Loss of normal regulation of NF-κB is a major contributor to a variety of diseases, including AID. In addition, a SNP (rs140490) in absolute LD (*r*^2^ = 1 and D' = 1) with miRSNP rs7444, was correlated with basal NF-kB activation in unstimulated B cells and monocytes (Lewis et al., 2015), suggesting an effect of miRSNP rs7444 in the regulation of this gene through miRNAs.

Moreover, the UBE2L3 protein has been described as involved in the cytotoxic function of NK cells, which is a key cell type in the innate immune response (Fortier and Kornbluth, 2006). Hence, it is conceivable that if any of the miRNAs predicted to target this gene are expressed in NK cells, they could regulate UBE2L3 expression in this cell type. Although it is not known if these four miRNAs are expressed in NK cells, according to miRmine database (Panwar et al., 2017), apart from miR-4741 that presents low levels in blood, the other miRNAs are highly expressed in blood and in plasma, and display normal levels of expression in T-cells (miRmine database Panwar et al., 2017; accessed July 2017). Functional NK cell-specific assays might help to confirm these predictions.

Another interesting example is the miRSNP rs907091 which is an eQTL for *IKZF3* and was previously reported as one of the possible causal SNPs of this autoimmune associated region (Farh et al., 2015). We found that the allele T of this SNP was predicted to affect the binding site of eight miRNAs to *IKZF3* mRNA. The same allele T had a protective effect in four AID (T1D, PBC, UC, and IBD). By integrating genotype and RNA-seq (mRNA and microRNA) data, we found miR-326 levels negatively correlated with *IKZF3* mRNA levels, in homozygous (T/T) individuals. Additionally, miR-326 displayed a high NBC Score (0.99) and is highly expressed in whole blood and plasma (miRmine database; Panwar et al., 2017, accessed July 2017), in agreement with the possible effect of this miRNA on *IKZF3* expression levels, which is also expressed in these tissues. The *IKZF3* gene (also known as *AIOLOS*) encodes an IKAROS family transcription factor involved in regulation of lymphocyte development (Cortes et al., 1999). Loss of IKZF3 in mice can prevent autoreactive B cells and decrease peritoneal, marginal and recirculating B cells (Wang et al., 1998; Cariappa et al., 2001), suggesting that low expression of IKZF3 could limit autoimmunity. This again matched our predictions, and eQTL results available in public databases. Furthermore, our results suggest that the downregulation of *IKZF3* showed by the eQTL results for blood (*P* = 3.9 × 10^−5^, Z-score = −4.11, Blood eQTL database Westra et al., 2013; accessed July 2017) could be caused through the interaction between miRNA and mRNA of this target gene. Functional experiments are necessary to confirm these results. We hypothesize that knockdown of miR-326 in blood cells would counteract the deregulation of *IKZF3*. Anyhow, this miRNA could be a candidate for future therapy of AID.

Although our correlation analyses did not show that levels of all 86 miRNAs correlated with expression levels of their respective predicted target genes, we still have found correlations with 9 target genes. Since the miRNA and mRNA expression analyses were performed on LCL, the lack of correlation could be explained by tissue-specific expression, either of the target genes, the miRNAs, or other interacting proteins and non-coding RNAs. Moreover, predicted tissue-specific target genes are typically expressed in the same tissue as the miRNA but at significantly lower levels than in tissues where the miRNA is not present (Sood et al., 2006). Therefore, levels of miRNAs and the cognate target mRNA would correlate only in certain tissues. In addition, under certain circumstances, the effect of an miRNA on its target can only be observed when the protein level is measured (Li et al., 2015; Seo et al., 2017).

Furthermore, after integrating our results with data of experimentally validated assays available in two databases, we found two genes and two miRNAs (Supplementary Table 3) reported as functionally validated in these databases. Interestingly, the rs3088081 miRSNP of the *SNAPC4* gene is an eQTL that affects interaction with only one miRNA (miR-3661). Although little is known about this gene, this observation indicates the need of further functional validation of our results in the specific cell types, which could confirm whether these miRNAs should be considered candidates to regulate AID associated genes.

GWAS has identified more than 250 susceptibility loci for AID (Ricaño-Ponce and Wijmenga, 2013). Many risk loci are shared between these diseases, which is consistent with them having an overlapping genetic background. The majority of genetic variants identified by GWAS were located in non-coding regions of the genome. A few AID studies reported association of differential susceptibility with SNPs at miRNA binding sites, such as rs3190930 in the *PTPRK* locus in CeD that alters the binding site for miR-1910 (Trynka et al., 2011). However, our study is the first that showed, in a systematic integrative manner, that immune associated non-coding SNPs could alter miRNAs binding sites. Overall, our integrative approach allowed us to find possible functional SNPs that were not described by the original GWAS. In addition, this approach could be extended to other complex diseases where GWAS summary statistics data are available. Thus, we highlighted miRNAs and genetic variarion at their binding sites as new candidates to be involved in the development of the AID.

## Author contributions

RdA study design, data analysis and manuscript writing. VC and MC study design and data analysis. MP-E study design, data interpretation and critical revision of the manuscript. All authors approved the version to be published.

### Conflict of interest statement

The authors declare that the research was conducted in the absence of any commercial or financial relationships that could be construed as a potential conflict of interest.
